# Crizotinib inhibits hyperpolarization-activated cyclic nucleotide-gated channel 4 activity

**DOI:** 10.1186/s40959-017-0020-z

**Published:** 2017-01-19

**Authors:** Zhushan Zhang, Tai-Qin Huang, Igor Nepliouev, Hengtao Zhang, Adam S. Barnett, Paul B. Rosenberg, Sai-Hong I. Ou, Jonathan A. Stiber

**Affiliations:** 1 Department of Medicine, Duke University Medical Center, Box 3126, Durham, NC 27710 USA; 2 Department of Medicine, University of California Irvine School of Medicine, Orange, CA 92868 USA

**Keywords:** Crizotinib, Bradycardia, HCN4, Sinoatrial node, Non-small cell lung cancer

## Abstract

**Background:**

Sinus bradycardia is frequently observed in patients treated with crizotinib, a receptor tyrosine kinase inhibitor used for the treatment of anaplastic lymphoma kinase (ALK)-rearranged non-small cell lung cancer (NSCLC). We investigated whether crizotinib could influence heart rate (HR) through direct cardiac effects.

**Methods:**

The direct effect of crizotinib on HR was studied using ECG analysis of Langendorff-perfused mouse hearts. The whole-cell patch clamp technique was used to measure the effects of crizotinib on the hyperpolarization-activated funny current, I_f_, in mouse sinoatrial node cells (SANCs) and hyperpolarization-activated cyclic nucleotide-gated channel 4 (HCN4) activity in HEK-293 cells stably expressing human HCN4.

**Results:**

Crizotinib resulted in a dose-dependent reduction in HR in isolated intact mouse hearts with a half maximal inhibitory concentration (IC50) of 1.7 ± 0.4 μmol/L. Because ECG analysis revealed that crizotinib (0–5 μmol/L) resulted in significant reductions in HR in isolated mouse hearts without changes in PR, QRS, or QT intervals, we performed whole-cell patch clamp recordings of SANCs which showed that crizotinib inhibited I_f_ which regulates cardiac pacemaker activity. Crizotinib resulted in diminished current density of HCN4, the major molecular determinant of I_f_, with an IC50 of 1.4 ± 0.3 μmol/L. Crizotinib also slowed HCN4 activation and shifted the activation curve to the left towards more hyperpolarized potentials.

**Conclusions:**

Our results suggest that crizotinib’s effects on HCN4 channels play a significant role in mediating its observed effects on HR.

## Background

Crizotinib is a first-in-class, orally available multi-targeted receptor tyrosine kinase (RTK) inhibitor for the treatment of anaplastic lymphoma kinase (*ALK*)-rearranged non-small cell lung cancer (NSCLC) that is locally advanced or metastatic [1]. In addition, crizotinib has demonstrated clinical activity in mesenchymal epithelial transition (MET) and ROS1-rearranged NSCLC and will likely play an expanding role in targeted therapy for NSCLC [2]. We previously identified asymptomatic marked sinus bradycardia (defined as heart rate ≤ 45) in three NSCLC patients treated with crizotinib without evidence of prolongation of PR or QT intervals [3]. Subsequently, we performed a retrospective analysis of 42 patients with NSCLC who were treated with crizotinib in clinical trials and found that a decrease in heart rate (HR) was common among patients treated with crizotinib [4]. Recently, a large retrospective analysis of HR changes during crizotinib therapy among patients with *ALK*‐positive NSCLC enrolled in the PROFILE 1005 and PROFILE 1007 clinical trials was analyzed [5]. For the 1053 patients analyzed, the mean maximum post-baseline HR decrease was 25 beats per minute (bpm) [5]. Overall, 441 patients (41.9%) had at least one episode of post-baseline sinus bradycardia (defined as HR < 60 bpm). The likelihood of experiencing sinus bradycardia was statistically significantly higher among patients with a pre-crizotinib treatment HR <70 bpm [5]. In another study, the effect of crizotinib on HR was also found to be serum concentration-dependent with an average 2.5 bpm decrease in HR for every 100 ng/mL increase of serum crizotinib concentration without clinically or statistically significant QT prolongation [6].

Although a decrease in HR is frequently observed in patients treated with crizotinib [5], the mechanism of the observed reduction in HR is unknown. It is also unclear if the decrease in HR is a direct cardiac effect since other indirect factors such as metabolic hormones and neurohormonal activity can have significant effects on HR, and crizotinib has been associated with endocrine effects such as lowering of testosterone levels in men [7, 8]. We, therefore, investigated whether crizotinib could influence HR through direct cardiac effects using a combination of approaches involving intact mouse heart physiology and cellular electrophysiology.

## Methods

### ECG analysis of Langendorff-perfused mouse hearts

Experiments involving mice were performed according to NIH policies outlined in the Guide for Care and Use of Laboratory Animals. Protocols for animal research were reviewed by the Institutional Animal Care and Use Committee of Duke University Medical Center. C57BL/6 J mice were obtained from The Jackson Laboratory (Bar Harbor, ME). Mice were anesthetized with isoflurane inhalation. After a surgical level of anesthesia was confirmed, a thoracotomy was performed and the heart removed quickly. Hearts were isolated in ice cold buffer and retrograde perfused in a Langendorff perfusion system with Krebs-Henseleit buffer, which contained (in mmol/L): NaCl 118, KCl 4.7, CaCl_2_ 2.0, MgSO_4_ 1.2, Na-EDTA 0.5, NaHCO_3_ 25, KH_2_PO_4_ 1.2, and glucose 11, and equilibrated with a mixed 95% O_2_ and 5% CO_2_. The procedure was completed within 2 minutes. Then the heart was placed in a small cradle filled with perfusion buffer. Four wire electrodes were positioned around the heart cradle in an approximated Einthoven configuration for recording of ECG signals, as previously described [9]. The heart was perfused with a constant flow rate of 2 ml/min at 37^o^ C. After stabilization for about 30 min, continuous ECG signals were recorded and analyzed with LabChart7 (ADInstruments Inc., Colorado Springs, CO) connected with an Oct Bio Amp and Power Lab 16/30 Data Acquisition System (AD Instruments Inc.). Crizotinib (0–5 μmol/L) was added to the perfusate and isolated hearts were allowed to equilibrate for 10–15 min after each change in crizotinib concentration. ECG parameters (PR, QRS and QT intervals) were measured as previously described [10]. QT intervals were shown, rather than the corrected QT interval (QTC), as changes in QTC reflected differences in heart rate rather than changes in ventricular depolarization/repolarization. For example, using the formula QTC = QT/(RR/100) ^1/2^ described for correcting QT intervals in mice [10], crizotinib (1 μmol/L) would result in a 28 ± 5% reduction in the QTC compared with untreated controls due to the increase in RR interval from 184 ± 5 to 378 ± 29 msec (reflecting a decrease in HR), even with no significant change in the QT interval (62.4 ± 2.6 vs. 64.3 ± 4.6 msec, control vs. crizotinib treated).

### Cell culture and immunoblotting

HEK-293 cells were obtained from ATCC (Manassas, VA) and were grown in Dulbecco’s modified media (DMEM) (Thermo Fisher, Waltham, MA) supplemented with 10% fetal bovine serum (Sigma, St. Louis, MO) and 100 IU/ml penicillin and 100 μg/ml streptomycin (Thermo Fisher). For creation of stable cell lines expressing human HCN4, HEK-293 cells were transfected using Fugene HD reagent (Promega, Madison, WI) with human HCN4/pcDNA3 plasmid [11, 12] linearized with the restriction enzyme Pvu I. Transfected cells were then maintained in selection media containing 0.4 mg/ml G418 (Thermo Fisher) to select for the transfected cell population. After 3–4 weeks of selection, individual clones were picked and maintained in G418 containing media. To confirm expression of HCN4 on a protein level, protein lysates from non-transfected control HEK-293 cells and HEK-293 cells with stable expression of human HCN4 were subjected to sequential immunoblotting for HCN4 and then tubulin. Rabbit anti-HCN4 IgG was obtained from EMD Millipore (Billerica, MA) and mouse anti-alpha-tubulin IgG was obtained from the Developmental Studies Hybridoma Bank at the University of Iowa (Iowa City, IA).

### Sinoatrial node (SAN) cell isolation

SAN cells were isolated as previously described [13]. The heart was excised quickly from a euthanized mouse and immersed into oxygenated Tyrode’s solution containing (in mmol/L): NaCl 140, KCl 5.4, MgCl_2_ 1.0, NaH_2_PO_4_ 0.33, CaCl_2_ 1.8, HEPES 5, and Glucose 10, at pH 7.4. To isolate single SANCs, the SAN was dissected out from surrounding atrial tissues and cut into several small pieces. The SAN was then enzymatically digested in Ca^2+^-free Tyrode’s solution containing BSA 0.2%, Type II Collagenase (Worthington, Lakewood, NJ) 0.25 mg/mL, and Elastase (Worthington) 0.2 mg/mL. The digestion step was carried out in a shaking incubator at 37 °C. The single SAN cells were released in modified Kraft-Brühe (KB) solution by gentle titration with a glass transfer pipette. KB solution included (in mmol/L): KCl 85, Potassium glutamate 20, KH_2_PO_4_ 20, Taurine 20, EGTA 0.5, Glucose 20, Creatine 5, Succinic acid 5, Pyruvic acid 5, MgSO_4_ 5, and HEPES 5, at pH 7.2. The isolated SANCs were stored in KB solution at 4 °C for at least 2 h before experiments. Those SANCs having a spindle-like shape and showing funny currents, I_f_, were chosen for patch clamp experiments.

### Cellular electrophysiology

Whole-cell patch clamp recordings of funny current, I_f_, and HCN4 currents were performed with a MultiClamp-700A amplifier, as described previously [13]. The currents were filtered at 2 kHz and sampled at a rate of 10 kHz. All experiments were carried out at room temperature, unless otherwise specified. Data were acquired and analyzed with pCLAMP software (Axon Instruments). Correction for liquid junction potentials was performed [14]. For funny current (I_f_) recording, single SAN cells were perfused with Tyrode’s solution containing 1 mmol/L BaCl_2_ and 2 mmol/L MnCl_2_ to block the inward rectifier (I_Kir_) and I_Ca_ currents. 4-Aminopyridine (2 mmol/L) was added to inhibit the endogenous transient potassium current (I_to_), which can overlap with and obscure I_f_ tail current recorded at +20 mV. The pipettes had resistances of 4–5 MΩ for experiments with SANCs and 2–4 MΩ for experiments with HEK-293 cells when filled with pipette solution containing (in mmol/L): 135 Potassium aspartate, 1 MgCl_2_, 1 CaCl_2_, 4 Mg-ATP, 0.4GTP, 6.6 Na_2_ phosphocreatine, 10 HEPES, and 10 EGTA with pH 7.2 (with KOH). The cell membrane potential was held at -35 mV, and I_f_ was elicited by a test pulse of −115 mV (2 s) from holding potential every 15 s. The cell membrane capacitance was monitored by a 10 msec voltage step of −10 mV from holding potential of −35 mV.

For HCN4 current (I_HCN4_) recordings in stably transfected HCN4-expressing HEK-293 cells, external solution was used containing (in mmol/L): NaCl 120, KCl 30, MgCl_2_ 1.0, CaCl_2_ 1.8, HEPES 5, and Glucose 5, with pH 7.4 (with NaOH), in which 1 mmol/L BaCl_2_ and 2 mmol/L 4-Aminopyridine were added to block I_Kir,_ and inhibit endogenous I_to_. The cell membrane was held at -10 mV. HCN4 current was then elicited by 15 s test pulses from −65 to -135 mV in 10 mV increments then back to +20 mV for 2 s to test tail current [15]. The cells were perfused with crizotinib (PF-02341066, Selleckchem, Houston, TX) which was then washed out. To control for potential effects of I_f_/I_HCN4_ run-down over time, the current recordings after washout were compared with those before and during crizotinib exposure.

I_f_/I_HCN4_ current amplitudes were determined by measuring the time-dependent inward currents that were sensitive to blockade with 2 mmol/L CsCl. The activation curve for I_HCN4_ was constructed on the relative membrane conductance by plotting normalized peak tail current amplitudes against the potential of test hyperpolarization, and then fit with a Boltzmann equation, as previously described [16]. Activation time constants were calculated by fitting experimental traces according to the single-exponential equation: I_f_ = A exp (−(*t-t*_*0*_)/_*Tau*_), where A is a fixed factor, t is the time, and t_0_ sets the onset of current activation-deactivation.

### Statistical analysis

Data are presented as means ± standard errors of the means. A Student’s *t* test was used for comparison of two groups, and a paired Student’s *t* test was used for comparisons before and after crizotinib exposure. Values of *p* of < 0.05 were considered significant.

## Results

### Crizotinib results in a dose dependent decrease in HR

**Fig. 1:**
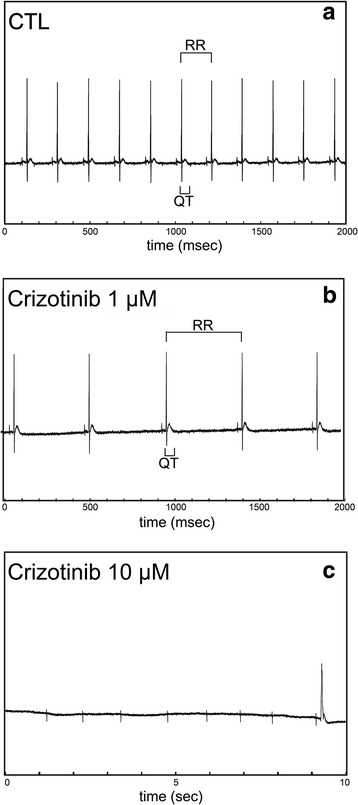
ECGs of isolated mouse hearts before and after crizotinib. **a** Representative ECG tracing, as described in Methods, recorded from an isolated mouse heart prior to exposure to crizotinib. RR and QT intervals are indicated as shown, and time is shown on x axis. (Note: time scale = 0–2000 msec for panels **a**-**b** and 0–10 s for panel **c**). **b**-**c** Representative ECG tracings recorded from the same isolated mouse heart as in (**a**) when exposed to 1 μmol/L (**b**) and 10 μmol/L crizotinib (**c**) respectively

**Fig. 2:**
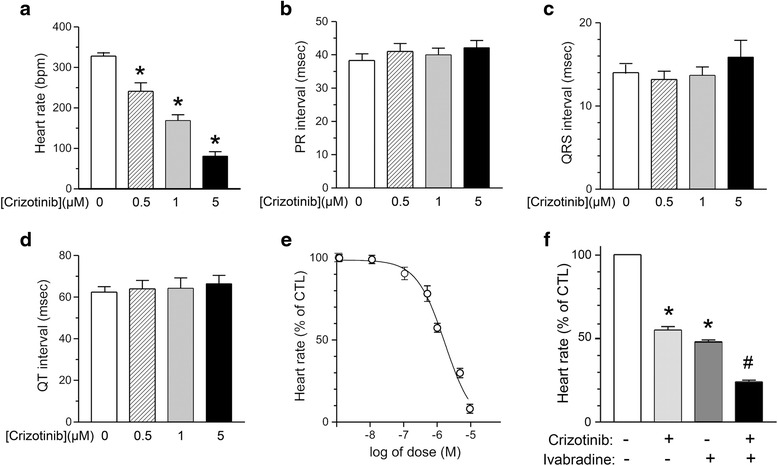
Effects of crizotinib on ECG parameters in isolated mouse hearts. **a** Isolated mouse hearts were exposed to increasing concentrations of crizotinib as shown (0 – 5 μmol/L, *n* = 6-11 for each concentration) and HR was measured by ECG. A significant reduction in HR was observed with increasing concentrations of crizotinib (*, compared with preceding concentration, *p* < 0.001). **b**-**d** Isolated mouse hearts were exposed to increasing concentrations of crizotinib (0–5 μmol/L, *n* = 6-11 for each concentration), and the following ECG parameters were measured as described in Methods: PR interval (**b**) QRS interval (**c**) and QT interval (**d**). **e** Dose–response curve showing effect of crizotinib on HR (expressed as % of unstimulated control) over a range of crizotinib concentrations (0–10 μmol/L, n = 6-11 for each concentration). The half maximal inhibitory concentration (IC50) of crizotinib on HR was observed to be 1.7 ± 0.4 μmol/L. **f** HR was measured by ECG in isolated mouse hearts under basal conditions (control, white bar) and following exposure to crizotinib (1 μmol/L, light grey bar, *n* = 11), ivabradine (3 μmol/L, dark grey bar, *n* = 5), or ivabradine plus crizotinib (3 μmol/L and 1 μmol/L respectively, black bar, *n* = 5). A significant reduction in HR was observed with either crizotinib or ivabradine (*, compared with CTL, *p* < 0.001) and with ivabradine plus crizotinib compared with ivabradine alone (#, compared with ivabradine alone, *p* < 0.001)

Mouse hearts were isolated and perfused as described in Methods in order to determine the direct effect of crizotinib on HR: excluding potential indirect cardiac effects that could be seen in an intact animal such as those mediated by metabolic hormones or neurohormonal activation. Increasing concentrations of crizotinib resulted in a dose-dependent decrease in HR as assessed by ECG analysis (Fig. 1a-b). This suggested that crizotinib had a direct effect on HR since potential indirect effects were eliminated by our isolated heart preparation. At high concentrations of crizotinib (≥10 μmol/L), we frequently observed high-degree AV block with persistent atrial activity (Fig. 1c), so concentrations (≥10 μmol/L) which were well above the range of clinically relevant concentrations, were not included in our subsequent analysis of ECG parameters. Crizotinib had a significant effect on HR at concentrations ≥ 0.5 μmol/L (Fig. 2a). The ECG parameters of PR interval (onset of atrial depolarization to onset of ventricular depolarization), QRS duration (duration of ventricular depolarization), and QT interval (duration of ventricular depolarization and repolarization) were observed over a range of physiologically relevant crizotinib concentrations 0–5 μmol/L, and no significant effects were observed (Fig. 2b-d). The half maximal inhibitory concentration (IC50) of crizotinib on HR was observed to be 1.7 ± 0.4 μmol/L (Fig. 2e). Because ECG analysis of isolated mouse hearts revealed that crizotinib (at concentrations ≤ 5 μmol/L) resulted in a decrease in HR in isolated intact mouse hearts without changes in PR, QRS, or QTC intervals, similar to as observed in human subjects [4, 6], we investigated whether crizotinib exerted its effects on HR through inhibition of the hyperpolarization-activated funny current, I_f_, in sinoatrial node cells (SANCs) which regulates cardiac pacemaker activity and thus HR. Ivabradine, a known inhibitor of the hyperpolarization-activated funny current, I_f_, at a concentration of 3 μmol/L which corresponds to the IC50 of ivabradine’s effect on I_f,_ [17], resulted in a similar reduction in heart rate in our isolated heart model as crizotinib (1 μmol/L). The addition of crizotinib (1 μmol/L) in the presence of ivabradine (3 μmol/L) resulted in an additive effect on reduction of heart rate compared with ivabradine alone (Fig. 2f).

### Crizotinib inhibits funny current in isolated mouse sinoatrial node cells

**Fig. 3:**
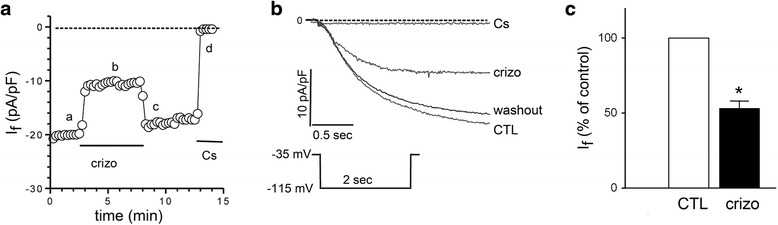
Crizotinib inhibits funny current (I_f_) in mouse sinoatrial node cells. The whole-cell patch clamp technique was used to measure the effects of crizotinib on I_f_ in isolated mouse sinoatrial node cells. **a** Representative tracing showing steady-state I_f_ over time (min) before (*a*) and during (*b*) perfusion of crizotinib (crizo, 1 μmol/L), after washout of crizotinib (*c*) and after addition of 2 mmol/L CsCl (*d*). Cell membrane potential was held at -35 mV, and I_f_ was elicited by a step to -115 mV for 2 s then return to holding potential (inset, panel **b**). Currents were normalized to membrane capacitance (pA/pF). **b** Representative single I_f_ tracings before (CTL), during perfusion with crizotinib (crizo), after washout of crizotinib (washout), and after addition of 2 mmol/L CsCl (Cs), corresponding to a-d in panel A. **c** Group mean values of I_f_ current expressed as % of control before (CTL) and after crizotinib (crizo, 1 μmol/L) (*n* = 6 for each group, *, *p* < 0.05)

We then investigated directly whether crizotinib could inhibit I_f_ in SANCs which regulates cardiac pacemaker activity and HR. The whole-cell patch clamp technique was used to measure I_f_ in mouse SANCs as described in Methods. Crizotinib (1 μmol/L) inhibited I_f_ which then returned to its pre-treatment level after crizotinib washout (Fig. 3a-b). Cesium chloride (CsCl, 2 mmol/L) resulted in complete inhibition of channel activity consistent with what would be expected for I_f_ (Fig. 3a-b). To eliminate potential confounding effects of I_f_ run-down over time, the current recordings after crizotinib washout were compared with those before crizotinib exposure and did not differ significantly (Fig. 3a-b). I_f_ current density after treatment with crizotinib (1 μmol/L) was 53 ± 5% that of control (Fig. 3c) (*n* = 6 for each group; *, *p* < 0.05).

### Crizotinib inhibits hyperpolarization-activated cyclic nucleotide-gated channel 4 (HCN4) activity

**Fig. 4:**
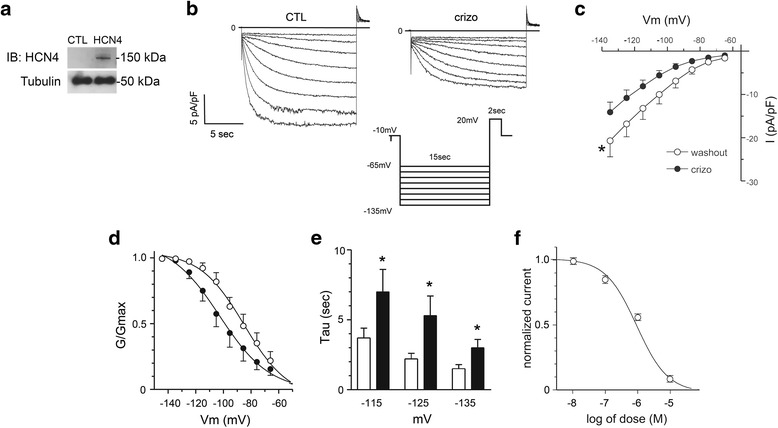
Crizotinib inhibits HCN4 channel activity. **a** Protein lysates from non-transfected control HEK-293 cells (CTL) and HEK-293 cells with stable expression of human HCN4 (HCN4) were subjected to sequential immunoblotting (IB) for HCN4 and then tubulin, as indicated. The whole cell patch-clamp technique was then used to assess HCN4 channel activity in HEK-293 cells with stable expression of human HCN4. **b** Representative traces of I_HCN4_ recorded in HCN4-expressing HEK-293 cells before (CTL) and during perfusion with crizotinib (crizo,1 μmol/L). I_HCN4_ was elicited by 15 s test pulses from a holding potential of -10 mV to test potentials ranging from −65 to -135 mV (in 10 mV increments) and then back to +20 mV for 2 s to test tail current, followed by return to holding potential (inset). **c**, Current–voltage (I-V) relationship for I_HCN4_ with crizotinib perfusion (crizo, filled circles) and after washout (open circles) (*n* = 9 per group). Current shown was normalized to membrane capacitance (pA/pF). A significant reduction in HCN4 current density was noted with crizotinib exposure at test potentials from −65 mv to −135 mV (*p* < 0.05, washout vs. crizotinib treated I-V curves by two-way ANOVA). **d** Effect of crizotinib on the voltage dependence of HCN4 activation. Activation curves were generated for I_HCN4_ with crizotinib perfusion (crizo, filled circles) and after washout (open circles). Crizotinib shifted the activation curve to the left towards more hyperpolarized potentials and shifted the average activation midpoint (V_0.5_) (V_0.5_ = −84 ± 2 vs. -102 ± 2 mV, washout vs. crizo, *p* < 0.05). **e** Average time constants (Tau) of HCN4 channel activation are plotted against test potentials with crizotinib perfusion (filled bars) and after washout (open bars) (*n* = 11 for each group). Crizotinib resulted in slower HCN4 channel activation at test potentials ≥ −115 mV (*, washout vs. crizo, *p* < 0.05). **f** I_HCN4_ (normalized to untreated controls) was measured at increasing crizotinib concentrations (0–10 μmol/L) in HCN4-expressing HEK-293 cells. A smooth curve was obtained by fitting the data with the Hill equation. Half-maximal inhibition concentration (IC50) was 1.4 ± 0.3 μmol/L (*n* = 4–18 at each concentration)

The molecular determinants for I_f_ are hyperpolarization-activated cyclic nucleotide-gated channels (HCNs) of which HCN4 is the predominant isoform in the sinoatrial node [18]. To avoid confounding effects of multiple ionic currents found in SANCs, we studied the effect of crizotinib on HCN4 activity using the whole-cell patch clamp technique in HEK-293 cells stably expressing human HCN4, as confirmed by immunoblotting (Fig. 4a). Crizotinib (1 μM) resulted in a significant decrease in HCN4 activity compared with pre-treatment control (Fig. 4b). As with measurements of I_f_, we compared current recordings after crizotinib washout with those prior to crizotinib exposure to eliminate potential confounding effects of I_HCN4_ run-down over time and saw no significant change (not shown). Comparison of the current–voltage (I-V) relationship after crizotinib washout with that during crizotinib exposure showed a significant reduction in HCN4 current density with crizotinib exposure over a range of test potentials from −65 mV to −135 mV (*, *p* < 0.05, washout vs. crizotinib treated I-V curves by two-way ANOVA) (Fig. 4c). At a test potential of −115 mV, crizotinib (1 μmol/L) resulted in a 40 ± 11% reduction in HCN4 current density (Fig. 4c). Crizotinib shifted the activation curve to the left towards more hyperpolarized potentials and shifted the average activation midpoint (V_0.5_, the voltage at which the tail current amplitude reached half maximal activation) from −84 ± 2 to −102 ± 2 mV (*, washout vs. crizotinib, *p* < 0.05) (Fig. 4d). In addition to affecting the voltage-dependence of HCN4 activation, crizotinib also affected the activation kinetics. Calculation of the average time constant (Tau) of channel activation revealed that crizotinib resulted in slower HCN4 channel activation at test potentials ≥ −115 mV (*, washout vs. crizo, *p* < 0.05) (Fig. 4e). The half-maximal inhibitory concentration (IC50) of crizotinib on HCN4 activity was 1.4 ± 0.3 μmol/L (*n* = 4-18 at each dose) (Fig. 4f).

## Discussion

Although we found that crizotinib inhibits HCN4 channel activity, it is a non-selective channel inhibitor [19, 20]. At high doses of crizotinib (≥10 μmol/L) we frequently observed high-degree AV block with persistent atrial activity (Fig. 1c) as has been observed in an inducible cardiac-specific HCN4 knockout mouse model [21], likely representing effects of crizotinib inhibition on HCN4 activity in the atrioventricular node and/or effects on other, perhaps multiple, cardiac channels. Because we chose to focus only on HCN4, the predominant HCN isoform in the SAN [18], we cannot rule-out the possibility that crizotinib may have differential effects on other HCN isoforms. Crizotinib has also been shown to inhibit cardiac delayed rectifier potassium current (I_Kr_), coded by the human ether-a-go-go-related gene (hERG) channel and has been shown to result in QT prolongation in isolated rabbit hearts at doses ≥ 10 μmol/L [19, 20]. We did not observe QT prolongation in our isolated mouse hearts likely due to the lower concentrations of crizotinib used in our studies as well as possible species differences. I_Kr_ is not prominent in adult mouse ventricular cardiomyocytes which limits the relevance of the mouse model for studying effects of crizotinib on QT prolongation in humans [22, 23]. Crizotinib has also been shown to inhibit CaV1.2 (IC50 = 3.5 μmol/L) and NaV1.5 channels (IC50 = 3.1 μmol/L) when expressed in heterologous cells [20]. Thus, the cardiac effects of crizotinib including its effects on HR are likely to represent effects on multiple cardiac ion channels.

The half maximal inhibitory concentration that we observed for HCN4 channels matches the IC50 that we observed for heart rate in our isolated heart preparations, suggesting that crizotinib’s effects on HCN4 channels contribute to its effects on HR. Moreover, the observed IC50 for crizotinib’s effects on HCN4 activity overlaps with the range of crizotinib plasma concentrations observed in patients [24]. Although human pharmacokinetic data have shown a maximum observed concentration, C_max_, of 65.5 ng/ml (~0.2 μmol/L) after a single 150 mg dose of crizotinib [25], steady state trough concentrations of crizotinib were found to range between 100–500 ng/ml (~0.2-1.1 μmol/L) for most patients treated with the typical crizotinib dose of 250 mg twice a day [2] and have been observed to be considerably higher in some patients [24, 26]. Thus, the crizotinib concentrations that we observed to have an inhibitory effect of HCN4 activity are clinically relevant. That a high percentage (~90%) of crizotinib is bound to plasma proteins [27], reducing free drug concentration, may explain why effects of crizotinib on HR are seen only in a subset of patients and are generally well tolerated [5].

The mechanism by which crizotinib inhibits HCN4 channels is beyond the scope of the current work but could involve either direct channel effects or indirect effects through crizotinib’s effects on multiple tyrosine kinases. Genistein, a tyrosine kinase inhibitor, was previously shown to inhibit I_f_ through a direct channel interaction [28]. Alternative possibilities include indirect effects of RTK inhibition such as potential inhibition of Src kinase activity by crizotinib, as Src tyrosine kinase can enhance HCN4 channel activity by directly phosphorylating the channel protein [15, 29]. That the bradycardia observed in patients treated with crizotinib, although generally asymptomatic, can be explained by a direct cardiac effect has important implications for patient safety. The current findings provide further justification for previous recommendations for patients to be monitored for the development of bradycardia while on crizotinib and for physicians to avoid concomitant use of common medications that can cause HR lowering and could potentially worsen the severity of bradycardia, such as beta-blockers and calcium channel blockers, during the course of crizotinib treatment [3, 4].

## Conclusions

We found that crizotinib results in a dose-dependent reduction in heart rate in isolated mouse hearts suggesting that crizotinib treatment results in sinus bradycardia through direct cardiac effects. We observed no significant changes in PR, QRS and QT intervals at doses which resulted in significant decreases in HR in isolated heart preparations. This suggested that crizotinib may result in a reduction in heart rate through effects on cardiac pacemaker activity. We then tested the effects of crizotinib in cardiac pacemaker cells of the SA node and found a significant reduction in I_f_ responsible for diastolic depolarization and spontaneous activity of these cells. Furthermore, crizotinib significantly inhibited HCN4 channel activity, the major molecular determinant of I_f_. Our results suggest that crizotinib’s effects on HCN4 channels play a significant role in mediating its observed effects on HR.

## Abbreviations

ALK: Anaplastic lymphoma kinase
crizo: Crizotinib
HCN4: Hyperpolarization-activated cyclic nucleotide-gated channel 4
HR: Heart rate
IC50: Half maximal inhibitory concentration
NSCLC: Non-small cell lung cancer
RTK: Receptor tyrosine kinase
SANC: Sinoatrial node cell
